# Bis(8-hy­droxy-2-methyl­quinolinium) bis­(pyridine-2,6-dicarboxyl­ato)cuprate(II) methanol monosolvate monohydrate

**DOI:** 10.1107/S160053681100674X

**Published:** 2011-02-26

**Authors:** Hossein Aghabozorg, Ahmad Gholizadeh, Masoud Mirzaei, Behrouz Notash

**Affiliations:** a Faculty of Chemistry, Islamic Azad University, North Tehran Branch, Tehran, Iran; b Department of Chemistry, School of Sciences, Ferdowsi University of Mashhad, Mashhad 917791436, Iran; c Department of Chemistry, Shahid Beheshti University, G. C., Evin, Tehran 1983963113, Iran

## Abstract

The title compound, (C_10_H_10_NO)_2_[Cu(C_7_H_3_NO_4_)_2_]·CH_3_OH·H_2_O was prepared by the reaction of copper(II) nitrate hexa­hydrate, 8-hy­droxy-2-methyl­quinoline, and pyridine-2,6-dicarb­oxy­lic acid in a 1:2:2 molar ratio in an aqueous solution. The geometry of the resulting CuN_2_O_4_ coordination can be described as distorted octa­hedral. In the crystal, there are several inter­molecular O—H⋯O, N—H⋯O and C—H⋯O hydrogen bonds. An intra­molecular N—H⋯O hydrogen bond occurs in one of the cations. Considerable π–π stacking inter­actions are also observed between the aromatic rings of the cations, with centroid–centroid distances of 3.4567 (13), 3.5342 (14), 3.6941 (14) and 3.4568 (13) Å. These non-covalent inter­actions connect the components, forming a three-dimensional supra­molecular structure.

## Related literature

For background to proton-transfer compounds, see: Aghabozorg *et al.* (2008 ▶). For examples of proton transfer from pyridine-2,6-dicarb­oxy­lic acid (pydcH_2_) to different amine base ligands, see: Eshtiagh-Hosseini *et al.* (2010*a* ▶,**b* ▶,c* ▶). 
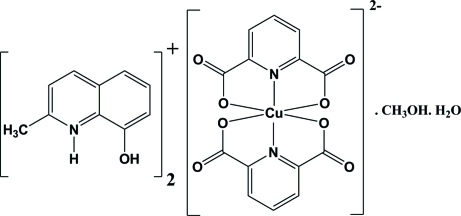


## Experimental

### 

#### Crystal data

(C_10_H_10_NO)_2_[Cu(C_7_H_3_NO_4_)_2_]·CH_4_O·H_2_O
*M* *_r_* = 764.20
Triclinic, 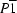

*a* = 10.116 (2) Å
*b* = 12.895 (3) Å
*c* = 14.816 (3) Å
α = 64.45 (3)°
β = 76.23 (3)°
γ = 83.74 (3)°
*V* = 1693.5 (8) Å^3^
*Z* = 2
Mo *K*α radiation
μ = 0.72 mm^−1^
*T* = 298 K
0.5 × 0.4 × 0.3 mm

#### Data collection

Stoe IPDS II diffractometer
Absorption correction: numerical (*X-SHAPE*; Stoe & Cie, 2005 ▶) *T*_min_ = 0.714, *T*_max_ = 0.803
18924 measured reflections
9061 independent reflections
7185 reflections with *I* > 2σ(*I*)
*R*_int_ = 0.028

#### Refinement

*R*[*F* ^2^ > 2σ(*F* ^2^)] = 0.038
*wR*(*F* ^2^) = 0.109
*S* = 1.01
9061 reflections
498 parameters
2 restraints
H atoms treated by a mixture of independent and constrained refinement
Δρ_max_ = 0.70 e Å^−3^
Δρ_min_ = −0.45 e Å^−3^

### 

Data collection: *X-AREA* (Stoe & Cie, 2005 ▶); cell refinement: *X-AREA*; data reduction: *X-AREA*; program(s) used to solve structure: *SHELXS97* (Sheldrick, 2008 ▶); program(s) used to refine structure: *SHELXL97* (Sheldrick, 2008 ▶); molecular graphics: *ORTEP-3 for Windows* (Farrugia, 1997 ▶); software used to prepare material for publication: *WinGX* (Farrugia, 1999 ▶).

## Supplementary Material

Crystal structure: contains datablocks I, global. DOI: 10.1107/S160053681100674X/vm2078sup1.cif

Structure factors: contains datablocks I. DOI: 10.1107/S160053681100674X/vm2078Isup2.hkl

Additional supplementary materials:  crystallographic information; 3D view; checkCIF report

## Figures and Tables

**Table 1:** Hydrogen-bond geometry (Å, °)

*D*—H⋯*A*	*D*—H	H⋯*A*	*D*⋯*A*	*D*—H⋯*A*
N3—H3*A*⋯O9	0.83 (3)	2.37 (2)	2.692 (2)	104.3 (17)
N3—H3*A*⋯O11	0.83 (3)	1.93 (3)	2.739 (3)	164 (2)
N4—H4*A*⋯O8^i^	0.95 (3)	1.87 (3)	2.723 (2)	149 (2)
O9—H9*A*⋯O5	0.78 (3)	1.79 (3)	2.563 (2)	176 (3)
O10—H10*A*⋯O4	0.87 (4)	1.70 (4)	2.555 (3)	167 (3)
O11—H11*A*⋯O1^i^	0.83 (4)	1.88 (4)	2.706 (3)	172 (4)
O12—H12*A*⋯O7	0.82 (3)	2.14 (3)	2.895 (3)	154 (4)
O12—H12*B*⋯O2^ii^	0.82 (4)	2.21 (4)	2.974 (3)	156 (4)
C10—H10⋯O3^iii^	0.93	2.55	3.177 (3)	125
C15—H15*C*⋯O2^i^	0.96	2.55	3.480 (3)	163
C17—H17⋯O6^iv^	0.93	2.29	3.185 (3)	161
C25—H25*C*⋯O8^i^	0.96	2.48	3.212 (3)	133
C27—H27⋯O2^v^	0.93	2.50	3.394 (3)	162

Symmetry codes: (i) 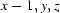; (ii) 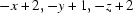; (iii) 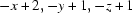; (iv) 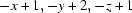; (v) 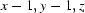.

**Fig. 1.**
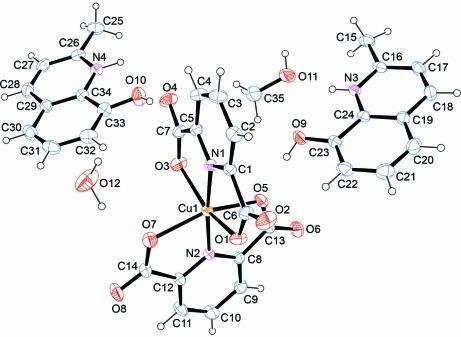
The molecular structure of the title compound with displacement ellipsoids drawn at 30% probability level.

**Fig. 2.**
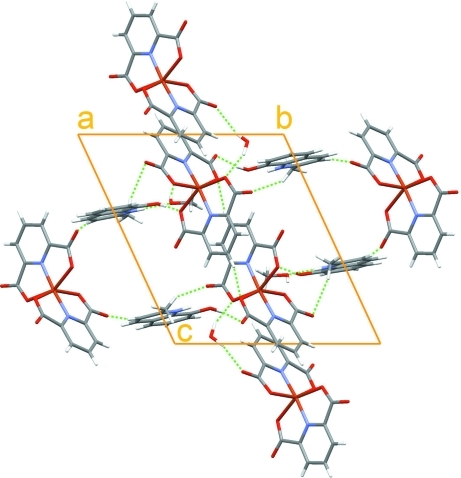
The packing diagram of the title compound. The intermolecular N—H···O, O—H···O and C—H···O hydrogen bonds are shown as green dashed lines.

**Fig. 3.**
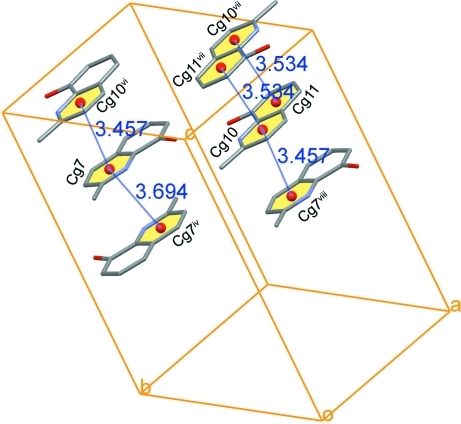
The packing diagram of the title compound showing π-π interactions between (8hmqH)+ fragments. Only (8hmqH)+ fragments without hydrogen atoms are shown for clarity. Cg7, Cg10 and Cg11 are the centroids of rings N3/C16—C19/C24, N4/C26—C29/C34 and C29—C34, respectively (symmetry codes: iv: 1 - x,2 - y,1 - z; vi: x,1 + y,z; vii:1 - x,-y,2 - z; viii: x,-1 + y,z).

## supplementary crystallographic information

### Comment

Our research focusses on water soluble proton transfer compounds as novel self-assembled systems that can function as suitable ligands in the synthesis of metal complexes. In this regard, we have reported examples of proton transfer from pyridine-2,6-dicarboxylic acid (pydcH_2_) to different amine base ligands (Eshtiagh-Hosseini *et al.* 2010*a*,**b*,c*). This has resulted in the formation of some novel proton transfer compounds based on carboxylic acid ligand derivatives (Aghabozorg *et al.* 2008).

The molecular structure of the title compound is presented in Fig. 1. In the title compound, Cu^II^ ion is six-coordinated by two pyridine-2,6-dicarboxylate, or (pydc)^2-^ groups and each (pydc)^2-^ ligand is coordinating through one pyridine N atom and two carboxylate O atoms. The atoms N1 and N2 of the two (pydc)^2-^ fragments occupy the axial positions, while atoms O1, O3, O5, and O7 form the equatorial plane [with Cu—O distances ranging from 2.1799 (16) to 2.2070 (16) Å]. The N1—Cu1—N2 angle [174.84 (5)°] deviates little from linearity. The O3—Cu1—O7 and O1—Cu1—O5 bond angles are equal to 91.22 (6)° and 94.25 (6)°, respectively. So the geometry of the resulting CuN2O4 coordination can be described as distorted octahedral. The packing diagram of the title compound is shown in Fig. 2. It is interesting to note that the space between the layers of [Cu(pydc)_2_]^2-^ units is occupied by (8hmqH)^+^ cations and uncoordinated water and methanol molecules, which the latter bridge the anionic and cationic units *via* hydrogen bonds (Fig 2 and Table 1). In the crystal structure, there are several intermolecular O—H···O, N—H···O, C—H···O and intramolecular N—H···O hydrogen bonds (Fig 2 and Table 1).

There are also extensive π–π interactions (Fig. 3) between the rings of (8hmqH)^+^ fragments with centroid–centroid distances for *Cg*7—*Cg*10^vi^, *Cg*10—*Cg*11^vii^, *Cg*7—*Cg*7^iv^ and *Cg*10—*Cg*7^viii^ equal to 3.4567 (13), 3.5342 (14), 3.6941 (14) and 3.4568 (13) Å, respectively, where *Cg*7, *Cg*10 and *Cg*11 are the centroids of N3/C16—C19/C24, N4/C26—C29/C34 and C29—C34, respectively (symmetry codes: iv: 1 - *x*,2 - *y*,1 - *z*; vi: *x*,1 + *y*,*z*; vii:1 - *x*,-*y*,2 - *z*; viii: *x*,-1 + *y*,*z*).

In the crystal packing a wide range of non-covalent interactions, consisting of hydrogen bonding and π ···π interactions plays an important role in the stabilization of the three-dimensional supramolecular network.

### Experimental

A solution of 8-hydroxy-2-methylquinoline (0.320 g, 2 mmol) in methanol (10 ml) and 2,6-pyridine dicarboxylic acid (0.170 g, 1 mmol) in methanol (10 ml) were mixed and stirrered until a clear solution was obtained. A solution of Cu(NO_3_)_2_.3H_2_0 (0.121 g, 0.5 mmol) in methanol (5 ml) was added to the acid-base mixture and stirred for 30 min. Crystals of the title compound suitable for X-ray analysis were obtained by slow evaporation after two weeks.

### Refinement

The hydrogen atoms bonded to O and N atoms were found in difference Fourier map and refined isotropically. The water hydrogen atoms were refined with *U*iso(H) = 1.2 *U*eq(O) and distance restraints of O—H 0.82 (3) and 0.82 (4) Å for H12A and H12B, respectively. The C—H protons were positioned geometrically and refined as riding atoms with C—H = 0.93 Å and *U*iso(H) = 1.2 *U*eq(C) for aromatic C—H groups, C—H = 0.96 Å and *U*iso(H) = 1.5 *Ueq*(C) for methyl group.

### Crystal data

**Table:**

(C_10_H_10_NO)_2_[Cu(C_7_H_3_NO_4_)_2_]·CH_4_O·H_2_O	*Z* = 2
*M**_r_* = 764.20	*F*(000) = 790
Triclinic, *P*1	*D*_x_ = 1.499 Mg m^−^^3^
Hall symbol: -P 1	Mo *K*α radiation, λ = 0.71073 Å
*a* = 10.116 (2) Å	Cell parameters from 9061 reflections
*b* = 12.895 (3) Å	θ = 2.3–29.2°
*c* = 14.816 (3) Å	µ = 0.72 mm^−^^1^
α = 64.45 (3)°	*T* = 298 K
β = 76.23 (3)°	Block, green
γ = 83.74 (3)°	0.5 × 0.4 × 0.3 mm
*V* = 1693.5 (8) Å^3^	

### Data collection

**Table:**

Stoe IPDS II diffractometer	9061 independent reflections
Radiation source: fine-focus sealed tube	7185 reflections with *I* > 2σ(*I*)
graphite	*R*_int_ = 0.028
Detector resolution: 0.15 mm pixels mm^-1^	θ_max_ = 29.2°, θ_min_ = 2.3°
rotation method scans	*h* = −13→13
Absorption correction: numerical (*X-SHAPE*; Stoe & Cie, 2005)	*k* = −17→17
*T*_min_ = 0.714, *T*_max_ = 0.803	*l* = −20→18
18924 measured reflections	

### Refinement

**Table:**

Refinement on *F*^2^	Primary atom site location: structure-invariant direct methods
Least-squares matrix: full	Secondary atom site location: difference Fourier map
*R*[*F*^2^ > 2σ(*F*^2^)] = 0.038	Hydrogen site location: inferred from neighbouring sites
*wR*(*F*^2^) = 0.109	H atoms treated by a mixture of independent and constrained refinement
*S* = 1.01	*w* = 1/[σ^2^(*F*_o_^2^) + (0.0728*P*)^2^] where *P* = (*F*_o_^2^ + 2*F*_c_^2^)/3
9061 reflections	(Δ/σ)_max_ = 0.002
498 parameters	Δρ_max_ = 0.70 e Å^−^^3^
2 restraints	Δρ_min_ = −0.45 e Å^−^^3^

### Special details

**Table:**

Geometry. All e.s.d.'s (except the e.s.d. in the dihedral angle between two l.s. planes) are estimated using the full covariance matrix. The cell e.s.d.'s are taken into account individually in the estimation of e.s.d.'s in distances, angles and torsion angles; correlations between e.s.d.'s in cell parameters are only used when they are defined by crystal symmetry. An approximate (isotropic) treatment of cell e.s.d.'s is used for estimating e.s.d.'s involving l.s. planes.
Refinement. Refinement of *F*^2^ against ALL reflections. The weighted *R*-factor *wR* and goodness of fit *S* are based on *F*^2^, conventional *R*-factors *R* are based on *F*, with *F* set to zero for negative *F*^2^. The threshold expression of *F*^2^ > σ(*F*^2^) is used only for calculating *R*-factors(gt) *etc*. and is not relevant to the choice of reflections for refinement. *R*-factors based on *F*^2^ are statistically about twice as large as those based on *F*, and *R*- factors based on ALL data will be even larger.

### Fractional atomic coordinates and isotropic or equivalent isotropic displacement parameters (Å^2^)

**Table:**

	*x*	*y*	*z*	*U*_iso_*/*U*_eq_	
Cu1	0.987953 (19)	0.531740 (17)	0.743974 (16)	0.03101 (7)	
N1	0.88383 (13)	0.54422 (11)	0.86756 (11)	0.0300 (3)	
N2	1.09007 (13)	0.53417 (11)	0.61416 (10)	0.0279 (3)	
O5	0.89361 (13)	0.67395 (12)	0.63268 (11)	0.0447 (3)	
O3	0.81065 (14)	0.42206 (13)	0.78562 (11)	0.0460 (3)	
O1	1.11637 (13)	0.64363 (13)	0.76480 (11)	0.0440 (3)	
O7	1.12019 (15)	0.38075 (12)	0.79369 (10)	0.0450 (3)	
O8	1.28336 (16)	0.28824 (13)	0.72425 (12)	0.0545 (4)	
O2	1.11205 (17)	0.73177 (15)	0.86636 (13)	0.0587 (4)	
O4	0.61123 (14)	0.37723 (14)	0.89804 (13)	0.0536 (4)	
C1	0.92907 (16)	0.61020 (14)	0.90270 (13)	0.0317 (3)	
C5	0.76455 (16)	0.48968 (14)	0.91421 (13)	0.0331 (3)	
C7	0.72560 (18)	0.42323 (15)	0.86147 (14)	0.0371 (4)	
C6	1.06461 (17)	0.66757 (15)	0.84037 (14)	0.0368 (4)	
C8	1.06465 (16)	0.61551 (13)	0.52592 (13)	0.0305 (3)	
C14	1.19898 (18)	0.36591 (15)	0.72033 (14)	0.0351 (3)	
C2	0.8541 (2)	0.62252 (17)	0.98891 (15)	0.0420 (4)	
H2	0.8858	0.6685	1.0132	0.050*	
C12	1.18579 (16)	0.45418 (14)	0.61431 (13)	0.0313 (3)	
O9	0.66369 (14)	0.76955 (12)	0.66892 (13)	0.0495 (4)	
O6	0.92311 (15)	0.77708 (13)	0.46303 (12)	0.0573 (4)	
C9	1.13573 (19)	0.61976 (16)	0.43302 (14)	0.0400 (4)	
H9	1.1166	0.6763	0.3723	0.048*	
C10	1.2365 (2)	0.53816 (19)	0.43151 (15)	0.0481 (5)	
H10	1.2865	0.5394	0.3696	0.058*	
N3	0.45289 (14)	0.91753 (13)	0.63308 (11)	0.0325 (3)	
C23	0.68490 (17)	0.87638 (16)	0.65494 (15)	0.0375 (4)	
C24	0.57362 (16)	0.95441 (15)	0.63553 (13)	0.0325 (3)	
C11	1.2618 (2)	0.45481 (18)	0.52344 (15)	0.0452 (4)	
H11	1.3295	0.3998	0.5239	0.054*	
C16	0.34540 (17)	0.98636 (16)	0.61397 (13)	0.0372 (4)	
C3	0.7318 (2)	0.5658 (2)	1.03830 (16)	0.0487 (5)	
H3	0.6807	0.5730	1.0965	0.058*	
C15	0.21709 (19)	0.9375 (2)	0.61694 (17)	0.0477 (5)	
H15A	0.2375	0.8892	0.5809	0.072*	
H15B	0.1575	0.9988	0.5850	0.072*	
H15C	0.1735	0.8929	0.6868	0.072*	
C4	0.68596 (19)	0.49856 (19)	1.00097 (15)	0.0452 (4)	
H4	0.6038	0.4598	1.0334	0.054*	
O10	0.52372 (17)	0.25204 (13)	0.83028 (15)	0.0587 (4)	
N4	0.38002 (15)	0.08098 (13)	0.84401 (12)	0.0349 (3)	
C21	0.8161 (2)	1.0311 (2)	0.64068 (19)	0.0548 (5)	
H21	0.8979	1.0560	0.6426	0.066*	
C26	0.30049 (18)	0.00004 (17)	0.85244 (14)	0.0400 (4)	
C19	0.58640 (19)	1.06926 (16)	0.61857 (14)	0.0389 (4)	
C33	0.58524 (19)	0.14932 (17)	0.85447 (16)	0.0415 (4)	
C25	0.1594 (2)	0.0336 (2)	0.83583 (19)	0.0551 (5)	
H25A	0.1027	0.0378	0.8961	0.083*	
H25B	0.1237	−0.0227	0.8219	0.083*	
H25C	0.1609	0.1072	0.7787	0.083*	
C17	0.3561 (2)	1.10237 (18)	0.59373 (15)	0.0460 (5)	
H17	0.2828	1.1521	0.5778	0.055*	
C22	0.80401 (19)	0.9164 (2)	0.65722 (18)	0.0490 (5)	
H22	0.8780	0.8667	0.6700	0.059*	
C34	0.50978 (17)	0.05901 (15)	0.86173 (13)	0.0337 (3)	
C18	0.4726 (2)	1.14235 (17)	0.59722 (16)	0.0475 (5)	
H18	0.4775	1.2189	0.5854	0.057*	
C20	0.7101 (2)	1.10638 (19)	0.62198 (18)	0.0508 (5)	
H20	0.7194	1.1819	0.6115	0.061*	
C32	0.7142 (2)	0.1243 (2)	0.87438 (17)	0.0510 (5)	
H32	0.7665	0.1821	0.8698	0.061*	
C29	0.56299 (19)	−0.05345 (16)	0.88863 (14)	0.0399 (4)	
C27	0.3516 (2)	−0.11168 (18)	0.87752 (16)	0.0474 (5)	
H27	0.2976	−0.1686	0.8825	0.057*	
C28	0.4794 (2)	−0.13769 (17)	0.89464 (16)	0.0478 (5)	
H28	0.5124	−0.2123	0.9106	0.057*	
C31	0.7666 (2)	0.0131 (2)	0.90131 (18)	0.0567 (6)	
H31	0.8536	−0.0018	0.9148	0.068*	
C30	0.6951 (2)	−0.0749 (2)	0.90864 (18)	0.0537 (5)	
H30	0.7330	−0.1482	0.9266	0.064*	
C13	0.95099 (17)	0.69800 (15)	0.53946 (15)	0.0371 (4)	
O11	0.37869 (17)	0.70218 (16)	0.6708 (2)	0.0806 (7)	
C35	0.4497 (3)	0.6032 (2)	0.6755 (3)	0.0786 (9)	
H35A	0.5415	0.6223	0.6374	0.118*	
H35B	0.4066	0.5655	0.6467	0.118*	
H35C	0.4505	0.5528	0.7456	0.118*	
O12	0.9690 (3)	0.1914 (2)	0.96545 (19)	0.0947 (8)	
H3A	0.446 (2)	0.8493 (19)	0.6449 (16)	0.035 (5)*	
H4A	0.342 (2)	0.156 (2)	0.8246 (19)	0.054 (7)*	
H9A	0.732 (3)	0.738 (3)	0.660 (2)	0.070 (9)*	
H10A	0.564 (3)	0.296 (3)	0.846 (2)	0.075 (9)*	
H11A	0.298 (4)	0.689 (3)	0.702 (3)	0.094 (11)*	
H12A	1.029 (3)	0.227 (3)	0.917 (2)	0.113*	
H12B	0.927 (4)	0.221 (3)	1.003 (3)	0.113*	

### Atomic displacement parameters (Å^2^)

**Table:**

	*U*^11^	*U*^22^	*U*^33^	*U*^12^	*U*^13^	*U*^23^
Cu1	0.02831 (10)	0.03006 (11)	0.03525 (12)	0.00009 (7)	−0.00293 (7)	−0.01629 (8)
N1	0.0281 (6)	0.0299 (6)	0.0327 (7)	−0.0011 (5)	−0.0045 (5)	−0.0146 (6)
N2	0.0274 (6)	0.0269 (6)	0.0296 (7)	0.0023 (5)	−0.0063 (5)	−0.0126 (5)
O5	0.0377 (6)	0.0417 (7)	0.0478 (8)	0.0125 (5)	−0.0056 (6)	−0.0172 (6)
O3	0.0490 (7)	0.0507 (8)	0.0423 (8)	−0.0129 (6)	0.0001 (6)	−0.0253 (6)
O1	0.0337 (6)	0.0550 (8)	0.0484 (8)	−0.0097 (5)	0.0033 (5)	−0.0304 (7)
O7	0.0557 (8)	0.0449 (7)	0.0320 (7)	0.0042 (6)	−0.0071 (6)	−0.0161 (6)
O8	0.0634 (9)	0.0454 (8)	0.0517 (9)	0.0267 (7)	−0.0230 (7)	−0.0183 (7)
O2	0.0605 (9)	0.0664 (10)	0.0627 (10)	−0.0269 (8)	−0.0034 (7)	−0.0386 (8)
O4	0.0424 (7)	0.0590 (9)	0.0635 (10)	−0.0217 (6)	0.0021 (7)	−0.0316 (8)
C1	0.0317 (7)	0.0315 (8)	0.0344 (8)	0.0016 (6)	−0.0082 (6)	−0.0160 (7)
C5	0.0301 (7)	0.0335 (8)	0.0335 (8)	−0.0038 (6)	−0.0032 (6)	−0.0130 (7)
C7	0.0377 (8)	0.0354 (9)	0.0378 (9)	−0.0072 (7)	−0.0048 (7)	−0.0149 (7)
C6	0.0349 (8)	0.0376 (9)	0.0413 (10)	−0.0057 (7)	−0.0065 (7)	−0.0194 (8)
C8	0.0291 (7)	0.0269 (7)	0.0348 (8)	0.0005 (6)	−0.0098 (6)	−0.0108 (6)
C14	0.0390 (8)	0.0329 (8)	0.0356 (9)	0.0057 (6)	−0.0129 (7)	−0.0153 (7)
C2	0.0466 (10)	0.0473 (10)	0.0410 (10)	0.0029 (8)	−0.0099 (8)	−0.0273 (9)
C12	0.0329 (7)	0.0307 (8)	0.0335 (8)	0.0061 (6)	−0.0093 (6)	−0.0167 (7)
O9	0.0309 (6)	0.0377 (7)	0.0792 (11)	0.0076 (5)	−0.0099 (7)	−0.0266 (7)
O6	0.0513 (8)	0.0447 (8)	0.0548 (9)	0.0159 (6)	−0.0168 (7)	−0.0022 (7)
C9	0.0460 (9)	0.0398 (9)	0.0307 (9)	0.0008 (7)	−0.0107 (7)	−0.0105 (7)
C10	0.0538 (11)	0.0567 (12)	0.0327 (9)	0.0082 (9)	−0.0030 (8)	−0.0229 (9)
N3	0.0306 (7)	0.0334 (7)	0.0327 (7)	0.0039 (5)	−0.0044 (5)	−0.0153 (6)
C23	0.0317 (8)	0.0381 (9)	0.0422 (10)	0.0035 (7)	−0.0053 (7)	−0.0186 (8)
C24	0.0308 (7)	0.0367 (8)	0.0302 (8)	0.0021 (6)	−0.0037 (6)	−0.0162 (7)
C11	0.0467 (10)	0.0489 (11)	0.0414 (10)	0.0176 (8)	−0.0082 (8)	−0.0249 (9)
C16	0.0310 (8)	0.0473 (10)	0.0292 (8)	0.0087 (7)	−0.0048 (6)	−0.0151 (7)
C3	0.0452 (10)	0.0655 (13)	0.0383 (10)	0.0003 (9)	0.0021 (8)	−0.0302 (10)
C15	0.0321 (8)	0.0600 (12)	0.0479 (11)	0.0052 (8)	−0.0096 (8)	−0.0207 (10)
C4	0.0350 (9)	0.0570 (12)	0.0390 (10)	−0.0082 (8)	0.0031 (7)	−0.0197 (9)
O10	0.0600 (9)	0.0412 (8)	0.0869 (13)	−0.0020 (7)	−0.0339 (9)	−0.0282 (8)
N4	0.0353 (7)	0.0378 (8)	0.0338 (8)	−0.0020 (6)	−0.0096 (6)	−0.0155 (6)
C21	0.0419 (10)	0.0660 (14)	0.0639 (14)	−0.0103 (9)	−0.0095 (9)	−0.0328 (12)
C26	0.0380 (9)	0.0491 (10)	0.0346 (9)	−0.0105 (7)	−0.0024 (7)	−0.0195 (8)
C19	0.0434 (9)	0.0373 (9)	0.0353 (9)	0.0007 (7)	−0.0040 (7)	−0.0171 (7)
C33	0.0429 (9)	0.0444 (10)	0.0429 (10)	−0.0047 (8)	−0.0140 (8)	−0.0201 (8)
C25	0.0367 (10)	0.0753 (15)	0.0601 (14)	−0.0075 (9)	−0.0082 (9)	−0.0341 (12)
C17	0.0470 (10)	0.0436 (10)	0.0400 (10)	0.0173 (8)	−0.0110 (8)	−0.0139 (8)
C22	0.0334 (9)	0.0571 (12)	0.0597 (13)	0.0040 (8)	−0.0121 (8)	−0.0275 (10)
C34	0.0360 (8)	0.0367 (8)	0.0297 (8)	−0.0025 (6)	−0.0090 (6)	−0.0136 (7)
C18	0.0566 (12)	0.0333 (9)	0.0472 (11)	0.0059 (8)	−0.0067 (9)	−0.0155 (8)
C20	0.0561 (12)	0.0436 (11)	0.0563 (13)	−0.0106 (9)	−0.0070 (10)	−0.0246 (10)
C32	0.0411 (10)	0.0705 (14)	0.0522 (12)	−0.0073 (9)	−0.0134 (9)	−0.0323 (11)
C29	0.0444 (9)	0.0401 (9)	0.0322 (9)	0.0020 (7)	−0.0087 (7)	−0.0128 (7)
C27	0.0549 (11)	0.0419 (10)	0.0439 (11)	−0.0167 (8)	−0.0006 (9)	−0.0181 (9)
C28	0.0617 (12)	0.0349 (9)	0.0419 (11)	−0.0013 (8)	−0.0065 (9)	−0.0138 (8)
C31	0.0369 (10)	0.0833 (17)	0.0556 (13)	0.0081 (10)	−0.0177 (9)	−0.0323 (12)
C30	0.0521 (11)	0.0576 (13)	0.0495 (12)	0.0180 (10)	−0.0198 (10)	−0.0203 (10)
C13	0.0302 (8)	0.0301 (8)	0.0455 (10)	0.0033 (6)	−0.0095 (7)	−0.0107 (7)
O11	0.0351 (8)	0.0562 (10)	0.139 (2)	−0.0101 (7)	0.0123 (10)	−0.0444 (12)
C35	0.0630 (15)	0.0560 (15)	0.107 (2)	−0.0065 (12)	0.0113 (15)	−0.0386 (16)
O12	0.125 (2)	0.0851 (16)	0.0707 (15)	−0.0470 (14)	0.0092 (13)	−0.0350 (12)

### Geometric parameters (Å, °)

**Table:**

Cu1—N2	1.9433 (15)	C15—H15A	0.9600
Cu1—N1	1.9461 (15)	C15—H15B	0.9600
Cu1—O5	2.1799 (16)	C15—H15C	0.9600
Cu1—O7	2.1880 (16)	C4—H4	0.9300
Cu1—O1	2.2018 (14)	O10—C33	1.337 (3)
Cu1—O3	2.2070 (16)	O10—H10A	0.87 (3)
N1—C1	1.336 (2)	N4—C26	1.332 (2)
N1—C5	1.341 (2)	N4—C34	1.375 (2)
N2—C12	1.335 (2)	N4—H4A	0.95 (3)
N2—C8	1.339 (2)	C21—C20	1.360 (3)
O5—C13	1.276 (2)	C21—C22	1.405 (3)
O3—C7	1.247 (2)	C21—H21	0.9300
O1—C6	1.268 (2)	C26—C27	1.396 (3)
O7—C14	1.260 (2)	C26—C25	1.492 (3)
O8—C14	1.234 (2)	C19—C20	1.407 (3)
O2—C6	1.230 (2)	C19—C18	1.412 (3)
O4—C7	1.248 (2)	C33—C32	1.379 (3)
C1—C2	1.386 (3)	C33—C34	1.413 (3)
C1—C6	1.520 (2)	C25—H25A	0.9600
C5—C4	1.385 (3)	C25—H25B	0.9600
C5—C7	1.516 (2)	C25—H25C	0.9600
C8—C9	1.374 (3)	C17—C18	1.357 (3)
C8—C13	1.517 (2)	C17—H17	0.9300
C14—C12	1.518 (2)	C22—H22	0.9300
C2—C3	1.380 (3)	C34—C29	1.409 (3)
C2—H2	0.9300	C18—H18	0.9300
C12—C11	1.380 (3)	C20—H20	0.9300
O9—C23	1.336 (2)	C32—C31	1.392 (3)
O9—H9A	0.78 (3)	C32—H32	0.9300
O6—C13	1.223 (2)	C29—C28	1.409 (3)
C9—C10	1.387 (3)	C29—C30	1.410 (3)
C9—H9	0.9300	C27—C28	1.355 (3)
C10—C11	1.385 (3)	C27—H27	0.9300
C10—H10	0.9300	C28—H28	0.9300
N3—C16	1.330 (2)	C31—C30	1.362 (4)
N3—C24	1.372 (2)	C31—H31	0.9300
N3—H3A	0.83 (2)	C30—H30	0.9300
C23—C22	1.375 (3)	O11—C35	1.378 (3)
C23—C24	1.421 (2)	O11—H11A	0.83 (4)
C24—C19	1.407 (3)	C35—H35A	0.9600
C11—H11	0.9300	C35—H35B	0.9600
C16—C17	1.404 (3)	C35—H35C	0.9600
C16—C15	1.487 (3)	O12—H12A	0.82 (3)
C3—C4	1.378 (3)	O12—H12B	0.82 (4)
C3—H3	0.9300		
			
N2—Cu1—N1	174.84 (5)	C16—C15—H15B	109.5
N2—Cu1—O5	77.26 (6)	H15A—C15—H15B	109.5
N1—Cu1—O5	98.34 (6)	C16—C15—H15C	109.5
N2—Cu1—O7	78.01 (6)	H15A—C15—H15C	109.5
N1—Cu1—O7	106.57 (6)	H15B—C15—H15C	109.5
O5—Cu1—O7	154.84 (6)	C3—C4—C5	118.75 (18)
N2—Cu1—O1	100.11 (6)	C3—C4—H4	120.6
N1—Cu1—O1	77.38 (6)	C5—C4—H4	120.6
O5—Cu1—O1	94.25 (6)	C33—O10—H10A	110.9 (19)
O7—Cu1—O1	94.57 (6)	C26—N4—C34	122.97 (16)
N2—Cu1—O3	104.79 (6)	C26—N4—H4A	115.8 (15)
N1—Cu1—O3	77.75 (6)	C34—N4—H4A	121.2 (15)
O5—Cu1—O3	90.63 (6)	C20—C21—C22	121.13 (19)
O7—Cu1—O3	91.22 (6)	C20—C21—H21	119.4
O1—Cu1—O3	155.09 (5)	C22—C21—H21	119.4
C1—N1—C5	120.77 (15)	N4—C26—C27	118.97 (17)
C1—N1—Cu1	119.89 (12)	N4—C26—C25	117.88 (18)
C5—N1—Cu1	119.29 (12)	C27—C26—C25	123.14 (19)
C12—N2—C8	120.70 (15)	C24—C19—C20	119.47 (17)
C12—N2—Cu1	119.15 (12)	C24—C19—C18	117.23 (18)
C8—N2—Cu1	120.15 (11)	C20—C19—C18	123.29 (19)
C13—O5—Cu1	114.39 (11)	O10—C33—C32	125.54 (19)
C7—O3—Cu1	112.59 (12)	O10—C33—C34	116.62 (17)
C6—O1—Cu1	113.61 (11)	C32—C33—C34	117.83 (19)
C14—O7—Cu1	113.22 (11)	C26—C25—H25A	109.5
N1—C1—C2	120.51 (16)	C26—C25—H25B	109.5
N1—C1—C6	114.34 (15)	H25A—C25—H25B	109.5
C2—C1—C6	125.15 (16)	C26—C25—H25C	109.5
N1—C5—C4	121.03 (17)	H25A—C25—H25C	109.5
N1—C5—C7	113.87 (15)	H25B—C25—H25C	109.5
C4—C5—C7	125.08 (16)	C18—C17—C16	120.42 (17)
O3—C7—O4	127.36 (18)	C18—C17—H17	119.8
O3—C7—C5	116.32 (15)	C16—C17—H17	119.8
O4—C7—C5	116.30 (17)	C23—C22—C21	121.22 (19)
O2—C6—O1	126.85 (17)	C23—C22—H22	119.4
O2—C6—C1	118.45 (17)	C21—C22—H22	119.4
O1—C6—C1	114.70 (15)	N4—C34—C29	119.13 (16)
N2—C8—C9	121.44 (15)	N4—C34—C33	119.13 (16)
N2—C8—C13	113.94 (15)	C29—C34—C33	121.73 (17)
C9—C8—C13	124.61 (15)	C17—C18—C19	120.87 (19)
O8—C14—O7	128.10 (17)	C17—C18—H18	119.6
O8—C14—C12	116.60 (16)	C19—C18—H18	119.6
O7—C14—C12	115.30 (14)	C21—C20—C19	119.6 (2)
C3—C2—C1	119.29 (18)	C21—C20—H20	120.2
C3—C2—H2	120.4	C19—C20—H20	120.2
C1—C2—H2	120.4	C33—C32—C31	120.5 (2)
N2—C12—C11	120.58 (16)	C33—C32—H32	119.8
N2—C12—C14	114.29 (15)	C31—C32—H32	119.8
C11—C12—C14	125.13 (15)	C28—C29—C34	117.42 (17)
C23—O9—H9A	111 (2)	C28—C29—C30	124.26 (19)
C8—C9—C10	118.74 (17)	C34—C29—C30	118.31 (19)
C8—C9—H9	120.6	C28—C27—C26	120.44 (19)
C10—C9—H9	120.6	C28—C27—H27	119.8
C11—C10—C9	119.10 (18)	C26—C27—H27	119.8
C11—C10—H10	120.5	C27—C28—C29	121.04 (19)
C9—C10—H10	120.5	C27—C28—H28	119.5
C16—N3—C24	122.87 (16)	C29—C28—H28	119.5
C16—N3—H3A	118.3 (14)	C30—C31—C32	122.37 (19)
C24—N3—H3A	118.8 (14)	C30—C31—H31	118.8
O9—C23—C22	125.73 (17)	C32—C31—H31	118.8
O9—C23—C24	116.22 (16)	C31—C30—C29	119.3 (2)
C22—C23—C24	118.04 (18)	C31—C30—H30	120.3
N3—C24—C19	119.72 (15)	C29—C30—H30	120.3
N3—C24—C23	119.76 (16)	O6—C13—O5	127.45 (17)
C19—C24—C23	120.52 (16)	O6—C13—C8	118.55 (18)
C12—C11—C10	119.43 (16)	O5—C13—C8	113.99 (15)
C12—C11—H11	120.3	C35—O11—H11A	112 (2)
C10—C11—H11	120.3	O11—C35—H35A	109.5
N3—C16—C17	118.85 (18)	O11—C35—H35B	109.5
N3—C16—C15	118.99 (18)	H35A—C35—H35B	109.5
C17—C16—C15	122.15 (16)	O11—C35—H35C	109.5
C4—C3—C2	119.63 (18)	H35A—C35—H35C	109.5
C4—C3—H3	120.2	H35B—C35—H35C	109.5
C2—C3—H3	120.2	H12A—O12—H12B	120 (4)
C16—C15—H15A	109.5		
			
O5—Cu1—N1—C1	−90.30 (13)	Cu1—N2—C12—C14	2.18 (19)
O7—Cu1—N1—C1	93.28 (13)	O8—C14—C12—N2	177.89 (16)
O1—Cu1—N1—C1	2.21 (12)	O7—C14—C12—N2	−1.2 (2)
O3—Cu1—N1—C1	−179.13 (13)	O8—C14—C12—C11	−1.1 (3)
O5—Cu1—N1—C5	87.16 (13)	O7—C14—C12—C11	179.80 (18)
O7—Cu1—N1—C5	−89.26 (13)	N2—C8—C9—C10	−0.5 (3)
O1—Cu1—N1—C5	179.67 (13)	C13—C8—C9—C10	−179.33 (18)
O3—Cu1—N1—C5	−1.67 (12)	C8—C9—C10—C11	0.3 (3)
O5—Cu1—N2—C12	−177.07 (13)	C16—N3—C24—C19	0.8 (3)
O7—Cu1—N2—C12	−1.75 (12)	C16—N3—C24—C23	−179.05 (16)
O1—Cu1—N2—C12	90.82 (13)	O9—C23—C24—N3	0.6 (3)
O3—Cu1—N2—C12	−89.82 (13)	C22—C23—C24—N3	−179.72 (18)
O5—Cu1—N2—C8	3.17 (12)	O9—C23—C24—C19	−179.23 (17)
O7—Cu1—N2—C8	178.49 (13)	C22—C23—C24—C19	0.4 (3)
O1—Cu1—N2—C8	−88.94 (13)	N2—C12—C11—C10	−1.2 (3)
O3—Cu1—N2—C8	90.42 (13)	C14—C12—C11—C10	177.78 (19)
N2—Cu1—O5—C13	−4.78 (13)	C9—C10—C11—C12	0.5 (3)
N1—Cu1—O5—C13	172.49 (13)	C24—N3—C16—C17	1.1 (3)
O7—Cu1—O5—C13	−15.6 (2)	C24—N3—C16—C15	−177.64 (17)
O1—Cu1—O5—C13	94.64 (14)	C1—C2—C3—C4	−0.4 (3)
O3—Cu1—O5—C13	−109.81 (14)	C2—C3—C4—C5	0.0 (3)
N2—Cu1—O3—C7	−171.70 (13)	N1—C5—C4—C3	0.7 (3)
N1—Cu1—O3—C7	3.68 (13)	C7—C5—C4—C3	−177.43 (18)
O5—Cu1—O3—C7	−94.71 (14)	C34—N4—C26—C27	−1.9 (3)
O7—Cu1—O3—C7	110.38 (14)	C34—N4—C26—C25	177.28 (18)
O1—Cu1—O3—C7	6.8 (2)	N3—C24—C19—C20	179.49 (17)
N2—Cu1—O1—C6	172.75 (13)	C23—C24—C19—C20	−0.6 (3)
N1—Cu1—O1—C6	−2.65 (13)	N3—C24—C19—C18	−1.4 (3)
O5—Cu1—O1—C6	94.96 (14)	C23—C24—C19—C18	178.43 (17)
O7—Cu1—O1—C6	−108.63 (14)	N3—C16—C17—C18	−2.3 (3)
O3—Cu1—O1—C6	−5.8 (2)	C15—C16—C17—C18	176.36 (19)
N2—Cu1—O7—C14	1.00 (13)	O9—C23—C22—C21	179.5 (2)
N1—Cu1—O7—C14	−176.57 (13)	C24—C23—C22—C21	−0.1 (3)
O5—Cu1—O7—C14	11.8 (2)	C20—C21—C22—C23	0.1 (4)
O1—Cu1—O7—C14	−98.39 (14)	C26—N4—C34—C29	0.9 (3)
O3—Cu1—O7—C14	105.86 (14)	C26—N4—C34—C33	−177.81 (18)
C5—N1—C1—C2	0.7 (2)	O10—C33—C34—N4	0.0 (3)
Cu1—N1—C1—C2	178.13 (13)	C32—C33—C34—N4	178.91 (18)
C5—N1—C1—C6	−179.02 (14)	O10—C33—C34—C29	−178.76 (18)
Cu1—N1—C1—C6	−1.60 (19)	C32—C33—C34—C29	0.2 (3)
C1—N1—C5—C4	−1.1 (3)	C16—C17—C18—C19	1.7 (3)
Cu1—N1—C5—C4	−178.51 (14)	C24—C19—C18—C17	0.2 (3)
C1—N1—C5—C7	177.25 (14)	C20—C19—C18—C17	179.2 (2)
Cu1—N1—C5—C7	−0.19 (19)	C22—C21—C20—C19	−0.3 (4)
Cu1—O3—C7—O4	174.11 (17)	C24—C19—C20—C21	0.6 (3)
Cu1—O3—C7—C5	−4.8 (2)	C18—C19—C20—C21	−178.4 (2)
N1—C5—C7—O3	3.6 (2)	O10—C33—C32—C31	178.4 (2)
C4—C5—C7—O3	−178.13 (18)	C34—C33—C32—C31	−0.4 (3)
N1—C5—C7—O4	−175.40 (16)	N4—C34—C29—C28	0.8 (3)
C4—C5—C7—O4	2.8 (3)	C33—C34—C29—C28	179.49 (19)
Cu1—O1—C6—O2	−176.75 (17)	N4—C34—C29—C30	−178.67 (17)
Cu1—O1—C6—C1	2.55 (19)	C33—C34—C29—C30	0.1 (3)
N1—C1—C6—O2	178.49 (17)	N4—C26—C27—C28	1.1 (3)
C2—C1—C6—O2	−1.2 (3)	C25—C26—C27—C28	−178.0 (2)
N1—C1—C6—O1	−0.9 (2)	C26—C27—C28—C29	0.6 (3)
C2—C1—C6—O1	179.41 (17)	C34—C29—C28—C27	−1.5 (3)
C12—N2—C8—C9	−0.1 (2)	C30—C29—C28—C27	177.9 (2)
Cu1—N2—C8—C9	179.63 (13)	C33—C32—C31—C30	0.4 (4)
C12—N2—C8—C13	178.78 (14)	C32—C31—C30—C29	−0.2 (4)
Cu1—N2—C8—C13	−1.46 (19)	C28—C29—C30—C31	−179.5 (2)
Cu1—O7—C14—O8	−179.14 (17)	C34—C29—C30—C31	−0.1 (3)
Cu1—O7—C14—C12	−0.18 (19)	Cu1—O5—C13—O6	−175.71 (17)
N1—C1—C2—C3	0.0 (3)	Cu1—O5—C13—C8	5.28 (19)
C6—C1—C2—C3	179.72 (18)	N2—C8—C13—O6	178.03 (17)
C8—N2—C12—C11	1.0 (3)	C9—C8—C13—O6	−3.1 (3)
Cu1—N2—C12—C11	−178.77 (14)	N2—C8—C13—O5	−2.9 (2)
C8—N2—C12—C14	−178.06 (14)	C9—C8—C13—O5	176.01 (17)

### Hydrogen-bond geometry (Å, °)

**Table:**

*D*—H···*A*	*D*—H	H···*A*	*D*···*A*	*D*—H···*A*
N3—H3A···O9	0.83 (3)	2.37 (2)	2.692 (2)	104.3 (17)
N3—H3A···O11	0.83 (3)	1.93 (3)	2.739 (3)	164 (2)
N4—H4A···O8^i^	0.95 (3)	1.87 (3)	2.723 (2)	149 (2)
O9—H9A···O5	0.78 (3)	1.79 (3)	2.563 (2)	176 (3)
O10—H10A···O4	0.87 (4)	1.70 (4)	2.555 (3)	167 (3)
O11—H11A···O1^i^	0.83 (4)	1.88 (4)	2.706 (3)	172 (4)
O12—H12A···O7	0.82 (3)	2.14 (3)	2.895 (3)	154 (4)
O12—H12B···O2^ii^	0.82 (4)	2.21 (4)	2.974 (3)	156 (4)
C10—H10···O3^iii^	0.93	2.55	3.177 (3)	125
C15—H15C···O2^i^	0.96	2.55	3.480 (3)	163
C17—H17···O6^iv^	0.93	2.29	3.185 (3)	161
C25—H25C···O8^i^	0.96	2.48	3.212 (3)	133
C27—H27···O2^v^	0.93	2.50	3.394 (3)	162

Symmetry codes: (i) *x*−1, *y*, *z*; (ii) −*x*+2, −*y*+1, −*z*+2; (iii) −*x*+2, −*y*+1, −*z*+1; (iv) −*x*+1, −*y*+2, −*z*+1; (v) *x*−1, *y*−1, *z*.
